# Organization, fine structure, and stereotaxic maps of the human Bed nucleus of the Stria terminalis

**DOI:** 10.1162/IMAG.a.1260

**Published:** 2026-06-15

**Authors:** Andrea Brandstetter, Sebastian Bludau, Hartmut Mohlberg, Philippe Massicotte, Alan C. Evans, Katrin Amunts

**Affiliations:** Institute of Neuroscience and Medicine (INM-1), Research Centre Jülich, Jülich, Germany; National Research Council of Canada (NRC), Ottawa, Canada; McGill Centre for Integrative Neuroscience, McGill University, Montreal, Canada; C. and O. Vogt Institute for Brain Research, Heinrich-Heine University, Düsseldorf, Germany

**Keywords:** Bed nucleus of the Stria terminalis, human brain anatomy, anxiety perception, cytoarchitectonic probabilistic maps, texture analysis, BigBrain model

## Abstract

The human Bed nucleus of the Stria terminalis is a basal forebrain structure and a key player in stress response and anxiety perception. It is a heterogenous structure and consists of distinct subdivisions. However, subdivisions are not visible in routine MR imaging due to low contrast and small size, which makes the exact assignment and identification in images of the living brain difficult or even impossible. Here, we mapped the Bed nucleus in serial, cell body-stained sections of 10 human brains in its full extent, resulting in 1130 annotations. Four subdivisions, a central, dorsal, medial, and a posterior part were identified. Texture analysis was applied to further characterize the subdivisions. Two sets of maps were generated: (1) Probabilistic cytoarchitectonic maps of the Bed nucleus in MNI space, which consider interindividual variability among the brains; (2) Ultra-high-resolution maps of the four subdivisions in the BigBrain 3D histology dataset, to capture complex shape and topology at microscopical level. The maps are openly available, to serve as anatomical reference to neuroimaging studies in healthy subjects and patients and inform modeling and simulation.

## 1 Introduction

The Bed nucleus of the Stria terminalis (BST) is a gray matter structure of the basal forebrain with a diameter of only a few millimeters. It was first described as „stria bed“ as part of the Caudate nucleus in the early 20th century (Johnston, 1923) and then as *Fundus subventricularis lateralis* and *Ncl. Subcaudatus* (Brockhaus, 1942). Decades later, the Bed nucleus was rediscovered and recognized as an independent structure using immunohistochemistry (Lesur et al., 1989; Martin et al., 1991; Walter et al., 1991). In the following years, structural and functional links between the Bed nucleus and the Amygdala resulted in the concept of the ‘extended Amygdala’ (Fox et al., 2015; Heimer & Van Hoesen, 2006; Heimer et al., 1999). The BST serves as a center of integration of limbic information; for an overview see Lebow and Chen (2016) or Shackman and Fox (2021).

**Fig. 1.**
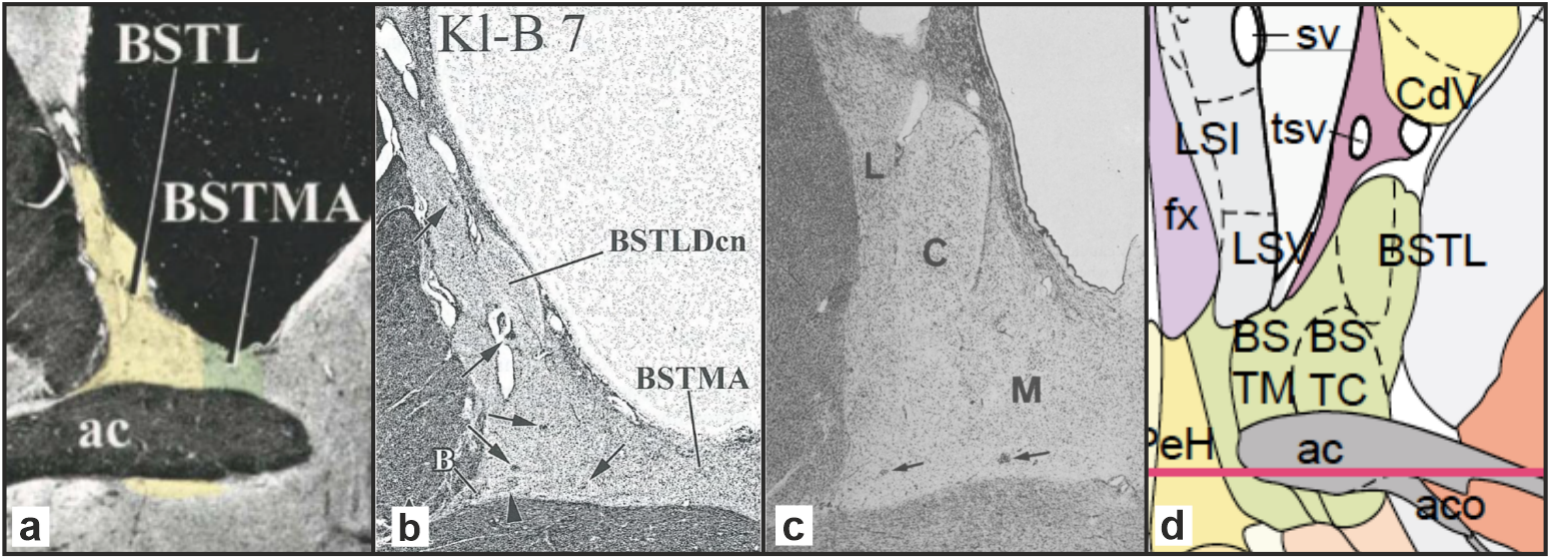
Subdivisions of the Bed nucleus at the level of the anterior commissure according to different authors. Heimer et al. (1999) distinguish (a) a lateral and medial part, and (b) provide a more detailed parcellation with a central part of the laterodorsal subdivision (BSTLDcn) and an anterior part of the medial BST (BSTMA). (c) Lesur et al. (1989) identified a lateral (L), central (C), and medial (M) subdivision of the BST. (d) Mai et al. (2007) with a similar parcellation scheme but with further, also unnamed delineations. Note: whereas a-c shows the BST in the left hemisphere, d depicts it in the right hemisphere.

Histological studies showed that the Bed nucleus is heterogenous and can be further subdivided (Fig. 1). Heimer and colleagues divided the BST into a lateral and medial part according to the ‘extended Amygdala’ concept, linking the BST and the Amygdala functionally (Fig. 1a). Their ‘Mini-Atlas’ is comprising a series of eight coronal sections of a single brain, each 50 µm thick, and with unknown intervals. In addition, they considered 11 subdivisions in the BST depicted on four additional sections of three further brains of unknown sex (Heimer et al., 1999): six subdivisions in the lateral BST, three in the medial, and two in the supracapsular part were labeled, but without providing their contour lines (Fig. 1b). Lesur et al. (1989) used immunocytochemistry to show a lateral, central, medial, and a fourth lateroventral subdivision in four representative levels, each 600 µm apart. This study was performed in histological sections (5 to 30 µm in thickness) of three male and two female brains (Fig. 1c). A more detailed atlas of the human brain features six named and further unnamed subdivisions of the BST across seven different levels (Mai et al., 2007). The maps are shown in coronal sections of intervals between 600 µm and 1.4 mm, based on a hemisphere of one male brain. Sections for the entire atlas were stained for myelin and cell bodies (Nissl, 20 µm). The delineation was based on the immunoreactivity of several neuropeptides (Mai et al., 2007) and the description by Brockhaus (1942). The oval central subdivision is delineated in most atlases (“BSTLDcn” in Fig. 1b, “C” in Fig. 1c, “BSTC” in Fig. 1d), allowing to compare this nucleus among different parcellation schemes. Lateral, dorsal, or medial subdivisions are also commonly used, often with various subsets of additional subdivisions (Fig. 1b–d). Other subdivisions such as fusiform or rhomboid nucleus are known in rodents but might refer to different names in human parcellation schemes (Walter et al., 1991).

Border definition of small subcortical structures in routine magnetic resonance (MR) images is often limited by resolution and contrast of this technique. Larger gray matter structures such as the Substantia nigra or the Red nucleus can easily be identified due to their size and the location in the white matter. The latter results in high contrast between neuron-rich regions and fiber tracts in MRI (Magnetic resonance imaging). Accordingly, the Bed nucleus is also well visible at the level of the decussating anterior commissure. However, even in high-field MRI it is almost impossible to identify BST borders at other levels and in respect of these circumstances many studies based on MRI have to rely on the appearance of anatomical landmarks or a certain length for anterior and posterior boundaries of the Bed nucleus (Avery et al., 2014; Neudorfer et al., 2020; Pauli et al., 2018; Sibbach et al., 2024; Theiss et al., 2017; Torrisi et al., 2019). Many of these studies rely on the detailed atlas of Mai et al. (2007), with limitations regarding unnamed subdivisions, unknown sources, and the non-probabilistic base of the atlas. Therefore, the BST can be easily under- or overestimated—even in high-field structural MRI.

Subdivisions of the BST and their connections have been widely investigated in mice and rats, in contrast to the one of humans (van de Poll et al., 2023). In short, the BST has afferent and efferent connections with the Amygdala and efferent connections with hypothalamic subdivisions and further to the brainstem (Dong et al., 2001; Erkan et al., 2024; Oler et al., 2017; Torrisi et al., 2015). It has been hypothesized that the subdivisions of the BST are a prerequisite to modulate anxiety: the anterior subdivision may be involved in two opposing circuits, one anxiolytic and one anxiogenic. Both circuits seem to be out of balance in anxiety or stress-related situations (Daniel & Rainnie, 2016). The central or oval subdivision of the BST is mainly involved in avoidance situations and stress response as it has been investigated in rats (Gungor & Pare, 2016). These findings might be applicable for humans as well (Avery et al., 2014); however, to what extent remains to be investigated, especially in the context of clinical applications: The Bed nucleus plays a key role in stress response, mood, and anxiety perception (Avery et al., 2016; Gungor & Pare, 2016; Knight & Depue, 2019; Shackman & Fox, 2021). Its functional role in these conditions makes the Bed nucleus an interesting region in the context of obsessive-compulsive disorder and depression besides the Amygdala, hippocampus or dorsomedial thalamus (Pandya et al., 2012). Deep brain stimulation (DBS) of the Bed nucleus has been performed in some case studies, and results seem to confirm a decrease of negative emotions (Winter et al., 2018), obsessions, and compulsions (Luyck et al., 2022; Luyten et al., 2016; Naesström et al., 2022) and general depressive and anxiety symptoms (Cassimjee et al., 2018; Luyten et al., 2016; Winter et al., 2018). Interestingly, studies do not mention exact coordinates, but rely on anatomical landmarks to approximate the BST (Cassimjee et al., 2018; Luyten et al., 2016; Naesström et al., 2022; Winter et al., 2018). The localization of the electrodes in DBS is usually guided by MRI and Computed Tomography (CT), mostly based on studies relying on visual inspection of these techniques (Neudorfer et al., 2020; Pauli et al., 2018; Sibbach et al., 2024; Theiss et al., 2017; Torrisi et al., 2019). The contrast and spatial resolution of the images, however, pose challenges with respect to the Bed nucleus. It can be easily found at the level of the anterior commissure, due to white matter surrounding the BST, while the nucleus is less visible in other places: The BST is directly adjacent to the Septal nuclei (anterior levels) and Hypothalamus (posterior levels); both are regions with no or low contrast in standard MR imaging. Moreover, the distinct subdivisions of the BST may also play a distinct role for positioning DBS electrodes, while they can also be distinguished by gold-standard staining only.

Therefore, the aim of this study was to analyze the microstructure of the BST, its subdivisions, extent, and localization; to map it cytoarchitectonically in a sample of 10 postmortem brains; to provide 3D maps that consider variations between brains; and to develop ultra-high-resolution maps in the BigBrain model (Amunts et al., 2013) to study the precise topography and localization of subdivisions.

## 2 Methods

### 2.1 Histological processing

Ten postmortem brains (Table 1), five male and five female, were obtained from the body donor program of the Anatomical Institute of the University of Düsseldorf, Germany (ethical vote of the medical faculty of the Heinrich-Heine-University Düsseldorf #4863). Age range was from 30 to 85 (mean: 57.7 years, SD = 19.2). Clinical records did not show any sign of neurological or neuropsychiatric diseases. The postmortem latency did not extend over 24 hours.

**Table 1.** Sample of postmortem brains.

Brain code	Age (yrs)	Gender	Cause of death	Fresh brain-weight [g]
**B01**	79	f	Bladder carcinoma	1350
**B03**	68	m	Cardiovascular disease	1360
**B05**	59	f	Cardiorespiratory insufficiency	1142
**B07**	37	m	Acute right heart failure	1437
**B08**	72	f	Renal failure	1216
**B10**	85	f	Mesenterial infarction	1046
**B12**	43	f	Pulmonary embolism	1198
**B13**	39	m	Drowning	1234
B20*	65	m	Cardiac insufficiency	1392
**B21**	30	m	Bronchopneumonia, Morbus Hodgkin	1409

The BigBrain (B20 (Amunts et al., 2013)) is marked by an asterisk.

The brains were fixed in either 4% buffered formalin or Bodian (a mixture of formalin, glacial acetic acid and ethanol) for more than 3 months. The fixed brains underwent MR scanning (1.5T field strength) to obtain an undistorted reference for subsequent 3D reconstruction (for a detailed description of the workflow see Amunts et al. (2020)). After dehydration, brains were embedded in paraffin, and serially sectioned in the frontal plane. Every 15th section was mounted on glass slides. One of the brains, B20, the BigBrain (Amunts et al., 2013), was processed with every single section (7404 sections), and used to analyze and map the extent of the Bed nucleus in every fifth section. The thickness of all sections was 20 µm. Cell bodies were stained using a silver method according to Merker (1983) and Uylings et al. (1999).

Digital scans of the cell body-stained sections were first acquired at 10 µm in-plane resolution, rescaled to 20 µm (Agfa DuoScan Flatbed Scanner), and then re-scanned at 1 µm in-plane resolution (TissueScope LE120, Huron Digital Pathology). Every 15th section was scanned. For the BigBrain-data, a high-resolution anatomical model of a human brain, each section was scanned and 3D-reconstructed (Amunts et al., 2013).

### 2.2 Identification of borders of the Bed nucleus and cytoarchitectonic mapping

The Bed nucleus and its subdivisions were identified by describing differences in the cytoarchitecture. Cytoarchitectonic criteria included the size (large-, medium- or small-sized), the shape (round, oval or multiform), and the density of neurons (dense or less dense) as well as cell distributions. Also, the orientation of neurons toward adjacent fibers and in some cases, the neuropil (translucent or a darker appearance) was used to identify subdivisions and to distinguish the Bed nucleus from neighboring structures. Criteria had to be recognizable on a minimum of five consecutive sections. Thus, the mapping of every 15th section led to a minimum extent of a subdivision of 1.5 mm in z-direction for each of the subdivisions and to a distance of 300 µm between two mapped sections. Mapping in the BigBrain on every 5th section further reduced the distance between two mapped sections to 100 µm. The mapping of all brains took place at a resolution up to 1 µm using a light microscope (Zeiss Axiolab 40, achroplan objectives, magnifications 4x and 10x) and annotations were immediately transferred to digitized images of 20 µm resolution, using the in-house software *OnlineSectionTracer* (Amunts et al., 2020). The nomenclature and compartmentalization of the BST mainly followed the cytoarchitectonic criteria of Heimer et al. (1999) but with some modifications and simplifications: The subdivisions were named without hierarchical order. Only regions that could be mapped in their full rostrocaudal extent were considered as distinct subdivisions. The juxtacapsular BST (BSTLJ after Heimer et al. (1999)) was seen on a few sections only and therefore could not be mapped in its full extent, but was included in the dorsal part of the BST. Furthermore, capsular or paracapsular parts were not distinguished for the same reasons.

### 2.3 Texture analysis using gray-level co-occurrence matrix (GLCM) in sequences of images of cell body-stained sections

To characterize and validate the four subdivisions in quantitative terms, a textural analysis was performed, using a Gray-Level Co-occurrence Matrix (GLCM). This matrix describes the frequency of the neighborhood relationships of adjacent gray value pairs (Haralick, 1979; Haralick et al., 1973). Texture analysis based on Haralick features has already been applied in different fields of research and industry (Fahmi et al., 2018; Nickolay & Vollmerhaus, 1988), in the medical field to detect bone fractures in x-ray images (Chai et al., 2011) and recently in high-resolution microscopic images of brain tissue (Kedo et al., 2024). The extracted features were adapted according to Löfstedt et al. (2019) incorporating the invariant Haralick features. This included a reduction of gray levels through quantization to create a normalized Gray Level Co-occurrence Matrix (GLCM). It encompasses all unique high-resolution annotations and extracted 21 invariant textural features, as opposed to the original four Haralick features.

Prior to the texture analysis, 8 bit images (256 gray values) of series of digitized histological sections were downloaded at microscopical resolution of 1 µm/px (TissueScope 120LE) and saved as whole-brain Bigtiff-files. In addition, the annotations from 10 different brains were saved as json-files. Altogether, 1130 Bed nucleus annotations have been saved and processed. The contour lines then served as regions of interest (ROIs). Small rectangular patches within these ROIs were analyzed to only use pixel pairs within the Bed nucleus for subsequent analysis.

To identify images that had to be excluded due to technical reasons, control images were generated using Matlab (The MathWorks, Inc., Natick, MA, USA). Possible exclusion criteria were inaccuracies in the registration of annotations caused by tissue artifacts or larger tissue tears within the BST, which would naturally lead to a change in the gray value composition of the analyzed image region. Blood vessels and other natural components identifiable within the BST were not explicitly masked. After this step, 1050 images were used for the analysis.

The Bed nucleus, as a three-dimensional structure, was examined in a sequence of histological 2D sections in the herein described analysis. This transition from 3D to 2D comes with the challenge that cross-sectioned structures could appear as several small parts on one histological section. In such a case, the extracted texture features described in the next section were combined with a weighted average according to the respective size of the annotations. This summary was made to consider the influence of possible stronger fluctuations of signals from small structures. Eight hundred seventeen distinct high-resolution annotations were created that were used for subsequent analyses.

**Fig. 2.**
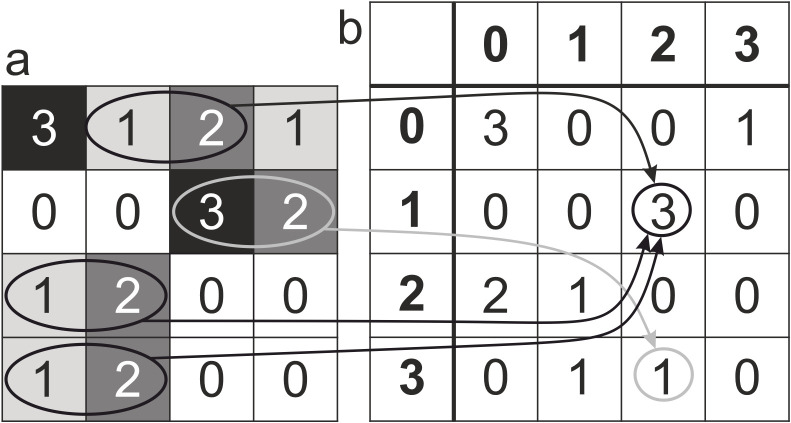
Example of a gray-level co-occurrence matrix. (a) Image of 4 × 4 pixel size, each with a specific gray value 0–3. (b) the matrix indicating the frequency of two neighboring pixels of the given image. The value 3 at position 1 × 2 (encircled in black) indicates that two adjacent pixels with value 1 and 2 occur three times in (a), whereas the value 1 at position 3–2 (encircled in gray) indicates that neighboring pixels 3–1 occur only once in this image.

Next, a mean histogram of all images was generated and applied on every single image to perform a histogram equalization compensating for slight intensity differences between different histological sections and brains. The following steps were in accordance with the publication of Löfstedt et al. (2019) using the invariant Haralick features. To give an example on how the matrix works: a value of 3 in the GLCM at position 1 × 2 indicates that the gray value 1 borders the gray value 2 three times in the original image (Fig. 2). The pairs of pixels 3-2 (Fig. 2a) occur in the given example only once and is represented in the matrix as value 1 (Fig. 2b), respectively. Additionally, the pixel neighborhood was determined in all directions and subsequently averaged.

These extracted 21 texture features were reduced with a principal component analysis (PCA) to four principal components describing the original 21 texture features that had an eigenvalue greater than one (Ringnér, 2008). Therefore, a correlation matrix was generated to plot the correlation coefficients between the 21 features. These features were weighted based on their influence on the results and step by step the number of components was reduced. The first component explains the majority of variance, the second a little bit less and so on. This step was calculated by a rotating varimax system until the final number of components was determined by the Kaiser–Meyer–Olkin measure. Thus, factors having an eigenvalue ≥1 in the correlation matrix were considered as a principal component (Guttman, 1954; Kaiser, 1960). Bartlett’s test of Sphericity was applied to test for any redundancies of the used variables (p ≤ 0.001).

Next, the Kruskal-Wallis test for non-parametric data was performed using SPSS (IBM SPSS Statistics for Windows, Armonk, NY, IBM Corp.) to test for differences between the subdivisions of the BST for each of the four main components. Post-hoc tests, using pairwise comparison (p ≤ 0.05) were calculated, to distinguish which subdivisions differed from each other and a Bonferroni correction was applied to correct for multiple tests. Additionally, a discriminant analysis based on the four main components of the extracted 21 invariant Haralick texture features was performed to further quantify and visualize the results.

### 2.4 Volumetric analysis

Volumes of the subdivisions of the 10 brains were calculated, considering the thickness of individual sections (20 µm), number of sections and the individual shrinkage factor (range 1.67 to 2.33). Shrinkage of the brains is inevitable due to histological processes and was described as the ratio of fresh brain weight prior fixation and the volume of the individual brains. The corrected volumes were calculated according to the Cavalieri principle (Amunts et al., 2007; Gundersen et al., 1988). Brain volumes were spatially normalized in order to compare brains of different sizes in a second step. Sex and hemispheric differences were tested by pair-wise permutation test using Matlab (The MathWorks, Inc., Natick, MA, USA) (Bludau et al., 2014). In addition, a linear regression analysis was performed to investigate a possible association between age and brain volume using Microsoft Excel (version 16.57).

Having annotated the Bed nucleus over its full extent, two different sets of maps were generated. The first dataset includes probabilistic maps of left and right BST, by combining the four subdivisions to a common data set. The second set of maps comprises detailed 3D reconstructions of the four individual subdivisions of the BST for each hemisphere using the BigBrain dataset.

### 2.5 Generation of cytoarchitectonic probability maps

While the subdivisions have been identified and labeled in all the brains, cytoarchitectonic probability maps were computed for the entire Bed nucleus of each hemisphere. This was following from the small extent of each subdivision of only a few millimeters, which was too close to the spatial resolution of the reference brain (see below). Each 15th section of the series was used for 3D reconstruction using linear and non-linear registration tools (Amunts et al., 2020). The calculated volumes were then transformed to the T1-weighted single-subject “Colin-27” template brain from the Montreal Neurological Institute (MNI) and the ICBM2009c Nonlinear Asymmetric space at the spatial resolution of 1 mm (Evans et al., 1992, 2012; Holmes et al., 1998) as two frequently used template brains. Both reference brains have a voxel size of 1 mm. The volumes were then superimposed in both anatomical reference spaces, and probabilistic maps were calculated. These maps show the probability of the Bed nucleus for each voxel of the reference brain in a range from 0% to 100 % and quantify therefore the interindividual variability among the brains in two reference spaces.

The maps are part of the Julich-Brain Atlas (Amunts et al., 2020) and openly available via the EBRAINS digital research infrastructure, available under https://ebrains.eu/service/human-brain-atlas/ and other resources and databases.

### 2.6 The BST and its subdivisions in high-resolution BigBrain space

To address the problem of spatial resolution, we used the BigBrain brain, with a 3D spatial resolution of 20 µm as an alternative reference (Amunts et al., 2013) to provide a high-resolution map of the Bed nucleus of the Stria terminalis. The mapping was performed in the 3D data set (https://bigbrainproject.org/) using the software suite Atelier 3D (https://mcin.ca/technology/visualization/atelier3d/). Atelier 3D is an interactive visualization and annotation tool, developed at the National Research Council of Canada that allows to simultaneously visualize and label all three orthogonal section planes of the BigBrain (Amunts et al., 2013; Borgeat et al., 2007). This enabled to capture the complex geometry of the nucleus and its subdivisions. Annotations in coronal, sagittal and horizontal planes were performed on every fifth section, resembling a distance between sections of 100 µm, and subsequently a surface mesh was computed. Atelier 3D was also used to visualize the 3D reconstruction of the four subdivisions as surfaces.

### 2.7 Comparison with previous maps

MRIcron (Rorden, 2025) was used to superimpose MR-based probabilistic maps from Sibbach et al. (2024) and Theiss et al. (2017) with recent cytoarchitectonic maps in the MNI Colin-27 single-subject reference space. The maps of Sibbach et al. (2024) are based manual delineations of the dorsal and ventral BNST in 7T images of 25 subjects. The maps of the Bed nucleus of Theiss et al. (2017) rely on 3T images of 10 subjects. The threshold was adjusted so that voxel intensities below 10% were excluded. A value of 100% represented the maximum intensity and the full extent of the structure. In the present analysis, only the dorsal subdivision of the Bed nucleus of the stria terminalis (dBNST) from the Sibbach et al. (2024) dataset was considered, since the region called the ventral subdivision (vBNST) did not meet our cytoarchitectonic criteria for the BST, but the hypothalamus instead.

## 3 Results

Macroscopically, the human Bed nucleus is located at the level of the decussation of the anterior commissure in the basal forebrain and is—according to its name—embedded between various structures: The Caudate nucleus represents the dorsal border at most levels. The ventral border is given by the Nucleus accumbens at rostral level, the anterior commissure at medial level and the Hypothalamus at caudal levels. The medial border is formed by the subependymal layer of the lateral ventricle and the Septal nuclei at rostral levels, the fornix and the Stria terminalis at middle levels and the Hypothalamus at caudal levels. The lateral border of the Bed nucleus is the internal capsule at all levels. The Caudate nucleus replaces the BST rostrally, while the Hypothalamus and Reticular nucleus of the thalamus form the caudal border.

### 3.1 Cytoarchitecture mapping

**Fig. 3.**
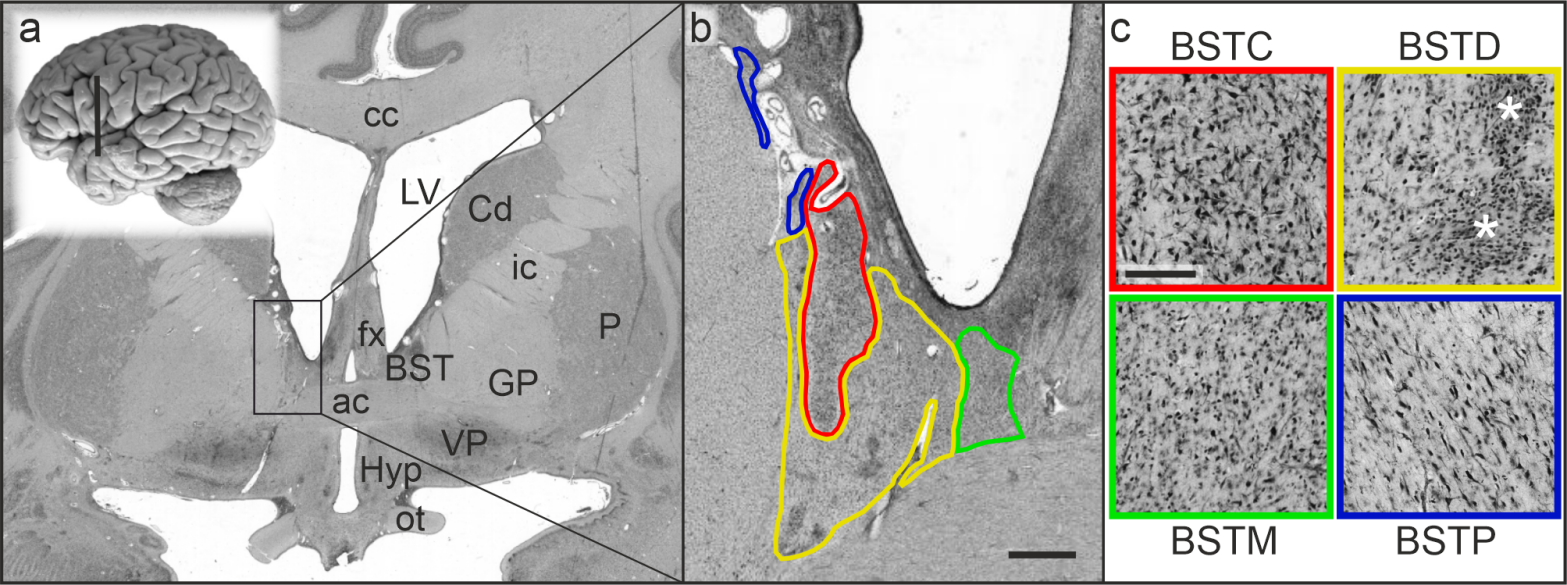
Cytoarchitecture of the Bed nucleus of the Stria terminalis, and its four subdivisions. (a) Coronal section at the level of the decussation of the anterior commissure with the Bed nucleus (BST), basal ganglia, hypothalamus and major surrounding structures, labeled in the right hemisphere. (b) Region of interest (rectangle in a) showing the Bed nucleus and its subdivisions: central part (BSTC) in red, dorsal part (BSTD) in yellow, medial part (BSTM) in green and posterior part (BSTP) in blue. Scalebar 1 mm. (c) Cytoarchitecture of the subdivisions at higher magnification: BSTC with triangular neurons, BSTD with mostly fusiform-shaped neurons and numerous interface islands (asterisks), that is, cell clusters comprising of very small, densely-packed granular neurons, BSTM with its characteristic heterogeneous cell population containing both densely-packed round neurons and larger multiform neurons and the BSTP containing fibers of the Stria terminalis and fusiform neurons oriented to these Stria fibers. Scalebar 200 µm. ac – anterior commissure, cc – corpus callosum, Cd – Caudate nucleus, fx – fornix, GP – Globus pallidus, Hyp – Hypothalamus, ic – internal capsule, ot – optic tract, LV – lateral ventricle, P – Putamen, VP – Ventral pallidum.

The Bed nucleus was mapped over its full extent, ranging from the head of the Caudate nucleus at rostral levels to the Reticular nucleus of the thalamus at caudal levels. Embedded between anterior commissure, fornix, lateral ventricle, Caudate nucleus, and internal capsule, the Bed nucleus can be easily annotated at the level of the anterior commissure (Fig. 3a). The main nomenclature of the BST followed the cytoarchitectonic criteria of Heimer et al. (1999). In contrast to this work, we did not distinguish between lateral and the medial subdivisions, nor between ventral or a supracapsular part.

The **central part of the Bed nucleus** (BSTC, Fig. 3) mainly consists of triangular and fusiform neurons. Neurons located dorsally in the BSTC were less densely packed than neurons located ventrally. Compared to the other subdivisions dendrites entering the soma can be clearly seen in the BSTC, whereas this subdivision has the least density of glial cells. The BSTC is surrounded by a thin band, scarcely populated by neurons and furthermore by another subdivision of the BST (see below). Only a small part of the BSTC reaches the surface of the Bed nucleus itself, one at the bottom of the BSTC, where it borders the anterior commissure. The other one at the mediodorsal end of the BSTC adjacent to the subventricular zone (svz). The svz is populated by densely packed neuroblasts and has a darker appearance in cell body-stained sections. The BSTC is almost completely surrounded by the **dorsal part of the Bed nucleus** (BSTD, Fig. 3). Neurons of the BSTD are fusiform, triangular, or oval in shape and of small or medium size. The neurons are smaller in size compared to those of the BSTC. Interface islands, that is, oval clusters of densely packed round, granular neurons are the most characteristic feature of this subdivision. The BSTD has larger and fewer neurons compared to the Nucleus accumbens. Also, due to the various shapes of the neurons in the BSTD, it gives the pattern a more heterogeneous appearance compared to the Nucleus accumbens, consisting of mostly round and oval neurons of the same size. The **medial part** (BSTM, Fig. 3) has both medium-sized and small neurons of various shapes and is the most heterogeneous subdivision of the Bed nucleus. Neurons lack of any orientation, glial cells are numerous. The BSTM is directly adjacent to the Septal nuclei and both structures share a similar cytoarchitectonic pattern. However, the neurons of the BSTM are smaller and have less medium-sized neurons compared to the septal region. On more caudal levels BSTD and BSTC are replaced by the **posterior part** (BSTP, Fig. 3), which forms the largest subdivision of the Bed nucleus. Its medium-sized neurons are loosely packed and mainly fusiform in shape. In posterior coronal sections they have a dorsoventral orientation parallel to the adjacent Stria terminalis, while there is no particular orientation in its ventral part. The BSTP has larger but fewer neurons compared to the BSTD, and smaller neurons compared to the anterior hypothalamic area.

### 3.2 Texture analysis of subdivisions of the Bed nucleus

**Fig. 4.**
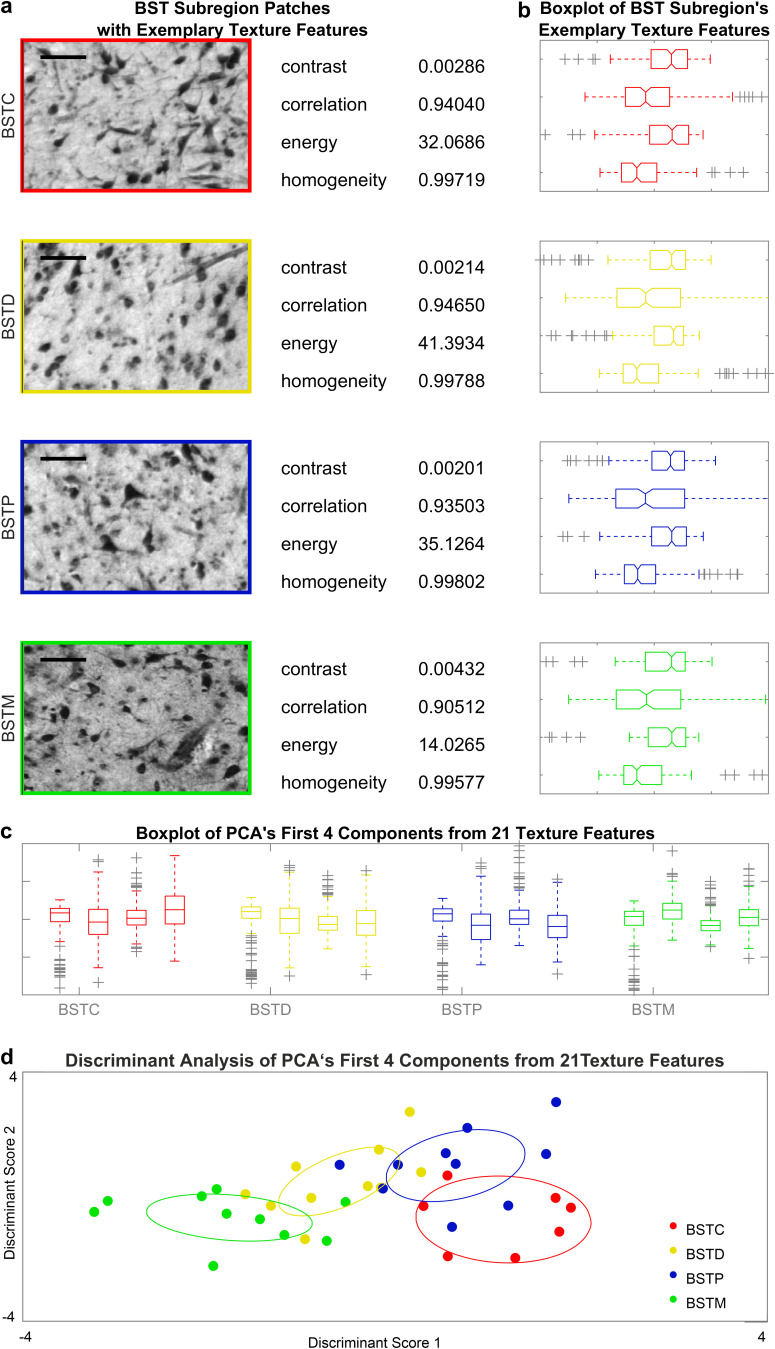
Results of the textural analysis. (a) Sample patches from the four BST subdivisions, displaying absolute values of exemplarily selected invariant Haralick textural features: contrast, correlation, energy, and homogeneity. Scalebar length 50 µm. (b) Z-score normalized boxplots showing median (central line), quartiles (box boundaries), and maximum values (whiskers) of the four chosen textural features across each subdivision. Crosses indicate outliers exceeding 1.5 times the interquartile range. (c) Z-score normalized boxplots of the four principal components derived from the 21 invariant Haralick features, presented for each subdivision. (b) and (c) were each calculated over the complete dataset of the 817 area-weighted annotations of the four subdivisions of the BST. (d) Canonical plot of Discriminant analysis visualizing the four distinct subdivisions of the Bed nucleus of the Stria terminalis based on the four extracted principal components. Filled circles refer to the mean values of all data points of each brain. Data points are color-coded to indicate group membership. Confidence ellipses, corresponding to each group, encircle the data points to illustrate the statistical spread and central tendency within the groups.

Individual image patches within the BST masks were combined and used for analysis in each subdivision of the BST and for the 21 textural features. Four exemplary patches and four of the 21 texture features are depicted in Figure 4a. A Principal Component Analysis (PCA) was applied to extract the most important independent factors prior to the textural analysis. The Kaiser-Meyer-Olkin measure of sampling adequacy was 0.771, and Bartlett’s test of Sphericity was significant (p < 0.001), indicating that correlations between items were sufficiently large for performing a PCA. Examination of Kaiser’s criteria and the scree-plot yielded empirical justification for retaining four factors with eigenvalues exceeding 1 which accounted for 91.8% of the total variance. The varimax-rotated four-factor solution yielded the most interpretable solution, and 50% loaded on the first factor. These calculations streamlined the gray-level co-occurrence matrix from a size of 817 × 21 (817 rows for each area-weighted annotation and 21 columns for each invariant Haralick feature) to a more compact 817 × 4 (817 rows for each annotation, with 4 columns representing the extracted principal components). Boxplots of four exemplary texture features for each of the BST subdivisions are shown in Figure 4b and for the four components in Figure 4c, respectively.

A Kruskal-Wallis test was conducted to determine whether there is a statistically significant difference between the four subdivisions regarding their texture. The results showed a significant difference for all four components (p < 0.01). Therefore, the Kruskal-Wallis test, as an omnibus test, proved that the null hypothesis could be rejected and a difference among the four subdivisions for all of the components can be assumed (p < 0.01). Bonferroni-corrected pairwise post hoc tests resulted in 16 of 24 significant differences (p < 0.05) among the four subdivisions (Table 2). Largest differences were identified between BSTC and BSTM as between BSTM and BSTP. Least differences, that is, only one significant result was observed between BSTC and BSTP. A discriminant analysis was calculated to visualize to what extent the four subdivisions were distinct to each other (Fig. 4d). Dorsal, medial, and posterior BST showed hardly any overlap; the central BST led to some overlap with the posterior ellipsoid.

**Table 2.**
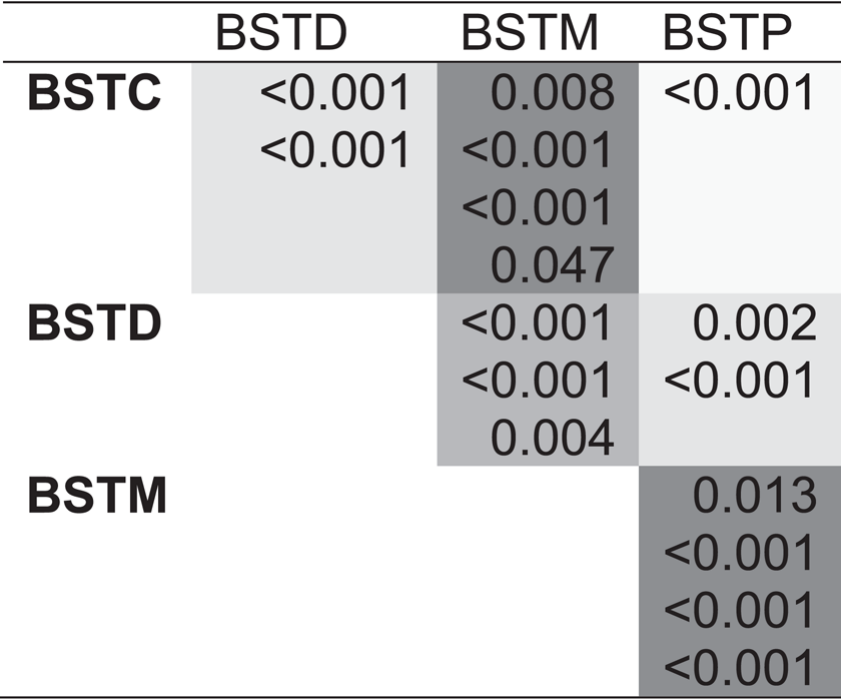
P-values of Bonferroni-corrected pairwise comparison post hoc tests.

Tests resulted in four significant pairwise comparisons between BSTC and BSTM as well as between BSTM and BSTP (dark gray). The comparison between BSTC and BSTP showed in only one significant result (light gray). Significance level 0.05.

### 3.3 Shape and extent of the Bed nucleus and its subdivisions

**Fig. 5.**
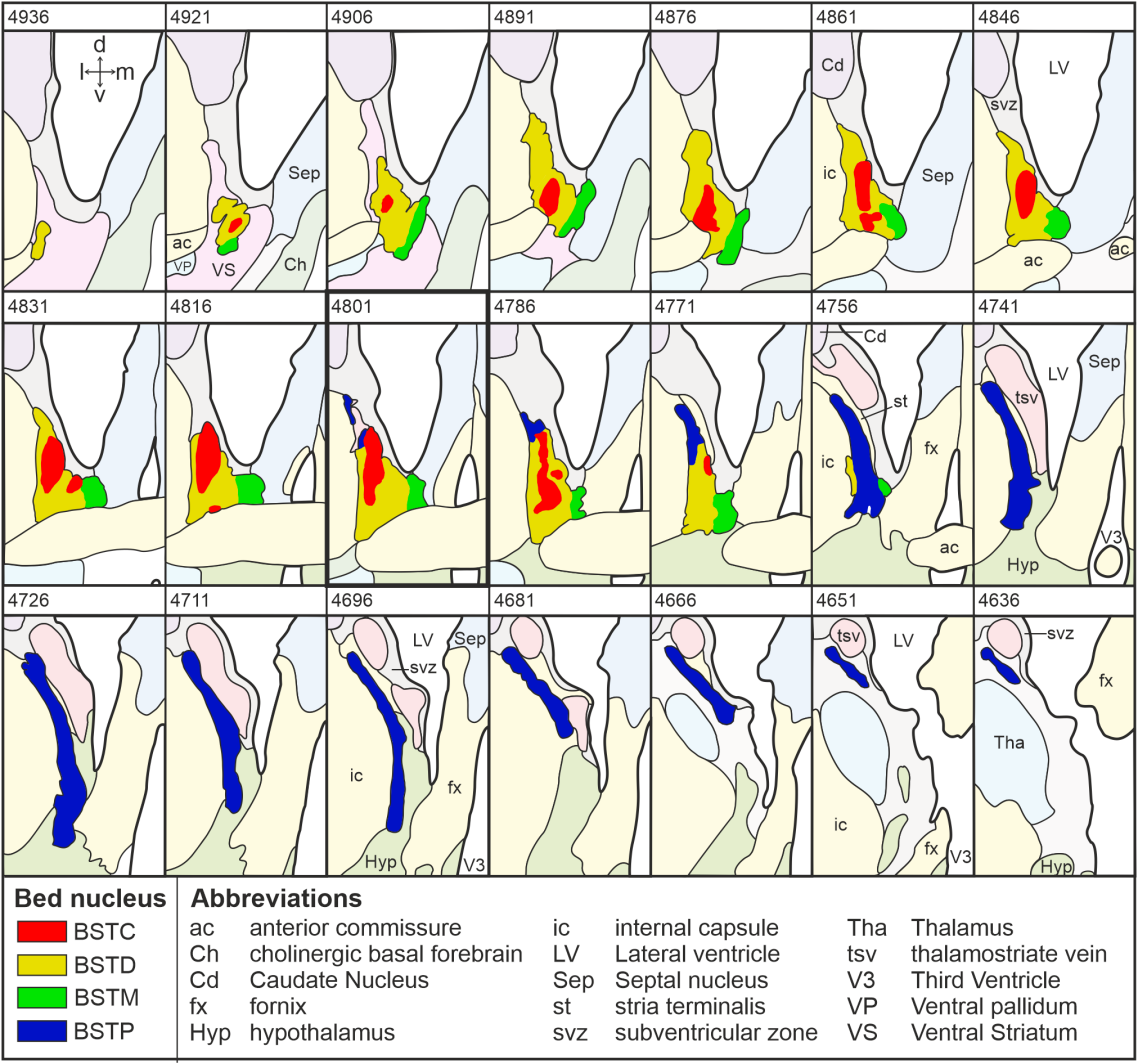
Rostro-caudal extent of the Bed nucleus on a series of coronal sections from rostral (section number 4936) to caudal (section number 4636) levels. Every 15th section is annotated, resulting in a distance of 300 µm between two sections. Section 4801 resembles Figure 3b. BSTC – central part, BSTD – dorsal part, BSTM – medial part and BSTP – posterior part.

The shape of the Bed nucleus of the Stria terminalis and each of its subdivisions vary depending on the level of coronal section (Fig. 5). At rostral levels, the Bed nucleus is more compact, it then changes to triangular shape at the level of the anterior commissure and finally gets elongated in shape at caudal levels. The subdivisions also change in shape when moving from rostral to caudal levels. The dorsal part (BSTD) is the one more compact at rostral levels and elongated in shape at its caudal end. It also flanks the anterior commissure ventrally and rostrally (Fig. 5, top and middle row). Also, it is almost fully covering the central part of the Bed nucleus (BSTC) with its oval shape at the level of the decussation of the anterior commissure (sections 4771–4921). The BSTC is narrowed towards the ventral and medial border of the BSTC. The smallest subdivision, the medial part of the Bed nucleus (BSTM) is located at the ventral tip of the frontal horn of the lateral ventricle, but not directly adjacent at the ventricular surface. It hardly varies in shape, is slightly elongated at the rostral end and mostly compact at the middle level. The posterior (BSTP) part of the Bed nucleus subsequently replaces the other three subdivisions just after the decussation of the anterior commissure. It seems to be the largest part of the Bed nucleus regarding its size and dorsoventral extent. It is visible on more than half of the coronal sections (Fig. 5, middle and bottom row).

### 3.4 Volumetric analysis

**Fig. 6.**
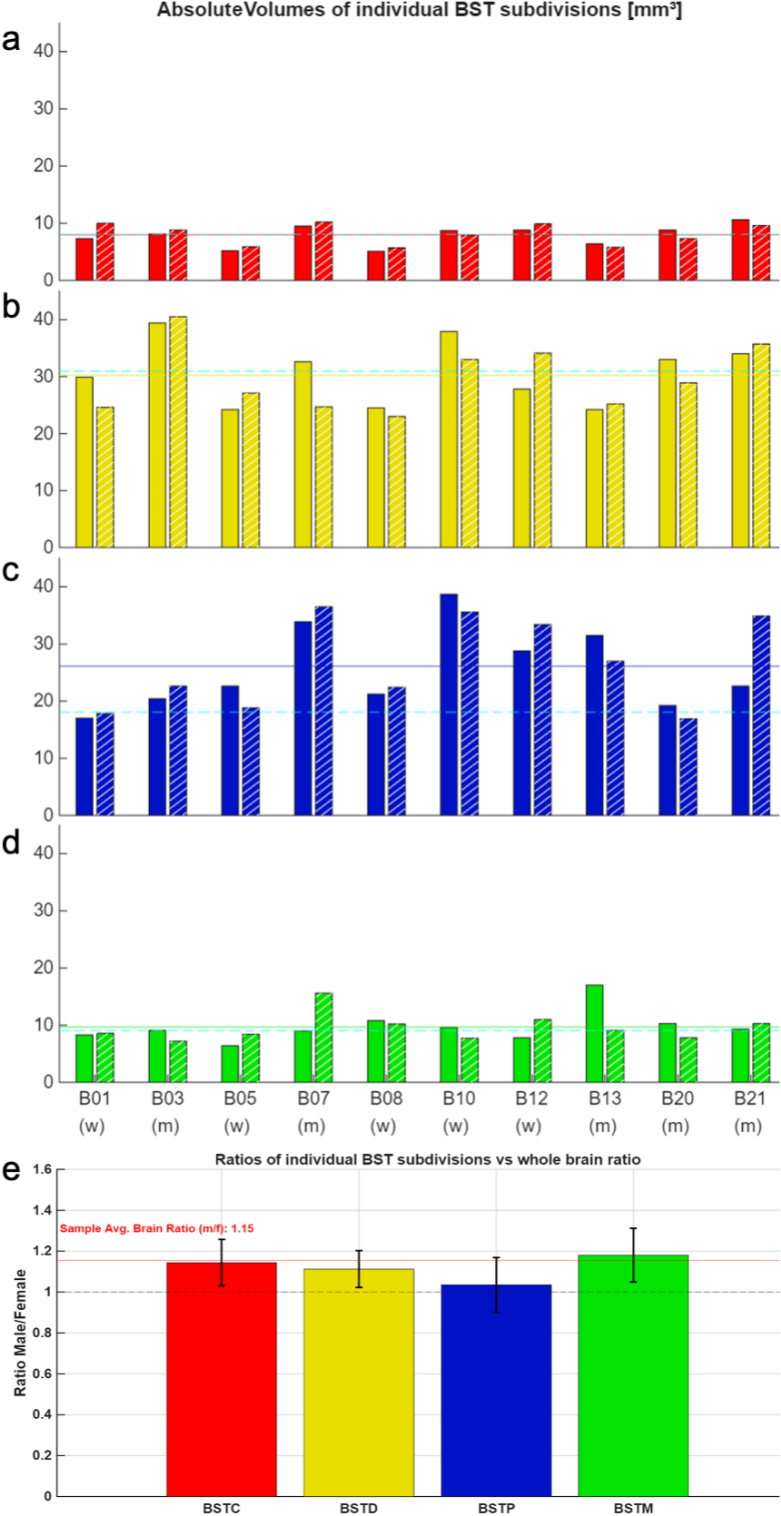
Absolute and Relative Volumes of BST subdivisions. (a) to (d) show the absolute volumes of the BST subdivisions (BSTC (red), BSTD (yellow), BSTM (green), BSTP (blue)) for both left (solid bars) and right (striped bars) brain hemispheres. The mean volume across all samples is indicated by a solid line, with a dashed line highlighting the left/right mean volume for the BigBrain (B20). (e) compares the male-to-female volume ratios of the above plotted BST subdivisions, showcasing the relative deviation through error bars. The solid red line represents the overall brain volume ratio between male and female sexes, while the black dashed line marks the parity ratio, illustrating a direct and concise comparison of BST subdivision volumes across sexes in relation to the total brain volume.

The Bed nucleus and its subdivisions were mapped in every 15th section, resulting in a distance of 300 µm between two sections and 1130 contours, that is, about 20 sections per brain and hemisphere. The shrinkage-corrected mean volume of the BST was 74.0 mm^3^ in each of the hemispheres (SD = 11.3 for left and 12.9 for right hemisphere). The mean volumes of the subdivisions range from 7.8 in the left BSTC to 30.7 in the left BSTD (Table 3). No sex or hemispheric differences could be shown within the normalized volumes (p > 0.05) for any of the subdivisions or the total volume and the utilized sample of 10 postmortem brains, with a male/female ratio of 1.15, exhibits a typical size ratio between the brain sizes of male and female body donors (Fig. 6; Table 3).

**Table 3.** Individual absolute and mean volumes for each of the subdivisions of the BST and standard deviation (SD) for the left and right hemisphere in mm^2^.

		B01 (F)	B03 (M)	B05 (F)	B07 (M)	B08 (F)	B10 (F)	B12 (F)	B13 (M)	B20* (M)	B21 (M)	Mean volumes [mm^2^] (SD)
BSTC	L	7.3	8.1	5.2	9.5	5.1	8.7	8.8	6.4	8.8	10.6	7.8 (1.8)
	R	10.0	8.8	5.9	10.12	5.7	7.9	9.9	5.8	7.3	9.6	8.1 (1.8)
BSTD	L	29.9	39.4	24.2	32.6	24.5	37.9	27.8	24.2	33.0	34.0	30.7 (5.6)
	R	24.6	40.5	27.1	24.7	23.0	33.0	34.1	25.2	28.9	35.7	29.6 (5.8)
BSTM	L	8.3	9.1	6.4	9.0	10.8	9.6	7.8	17.0	10.3	9.3	9.7 (2.8)
	R	8.6	7.2	8.4	15.6	10.2	7.7	11.0	9.1	7.8	10.3	9.5 (2.4)
BSTP	L	17.0	20.4	22.6	33.9	21.2	38.7	28.8	31.5	19.2	22.6	25.6 (7.2)
	R	17.8	22.6	18.8	36.5	22.4	35.6	33.4	27.0	16.9	34.9	26.5 (7.9)
Total Volume	L	62.5	77.1	58.4	85.1	61.6	95.0	73.2	79.2	71.3	76.5	73.9 (11.3)
	R	61.1	79.1	60.2	87.0	61.3	84.2	88.4	67.1	61.0	90.4	73.9 (12.9)

(F) female, (M) male, *BigBrain.

A linear regression was conducted to study the possible relationship between age and volume of the BST and its subdivisions. No significant relationship was found: p = 0.733, R² = 0.0153, 95% CI -0.547, 0.402 for the left BST; and p = 0.174, R² = 0.2171, 95% CI -0.8, 0.172 for the right BST.

### 3.5 Probabilistic maps in stereotaxic space

**Fig. 7.**
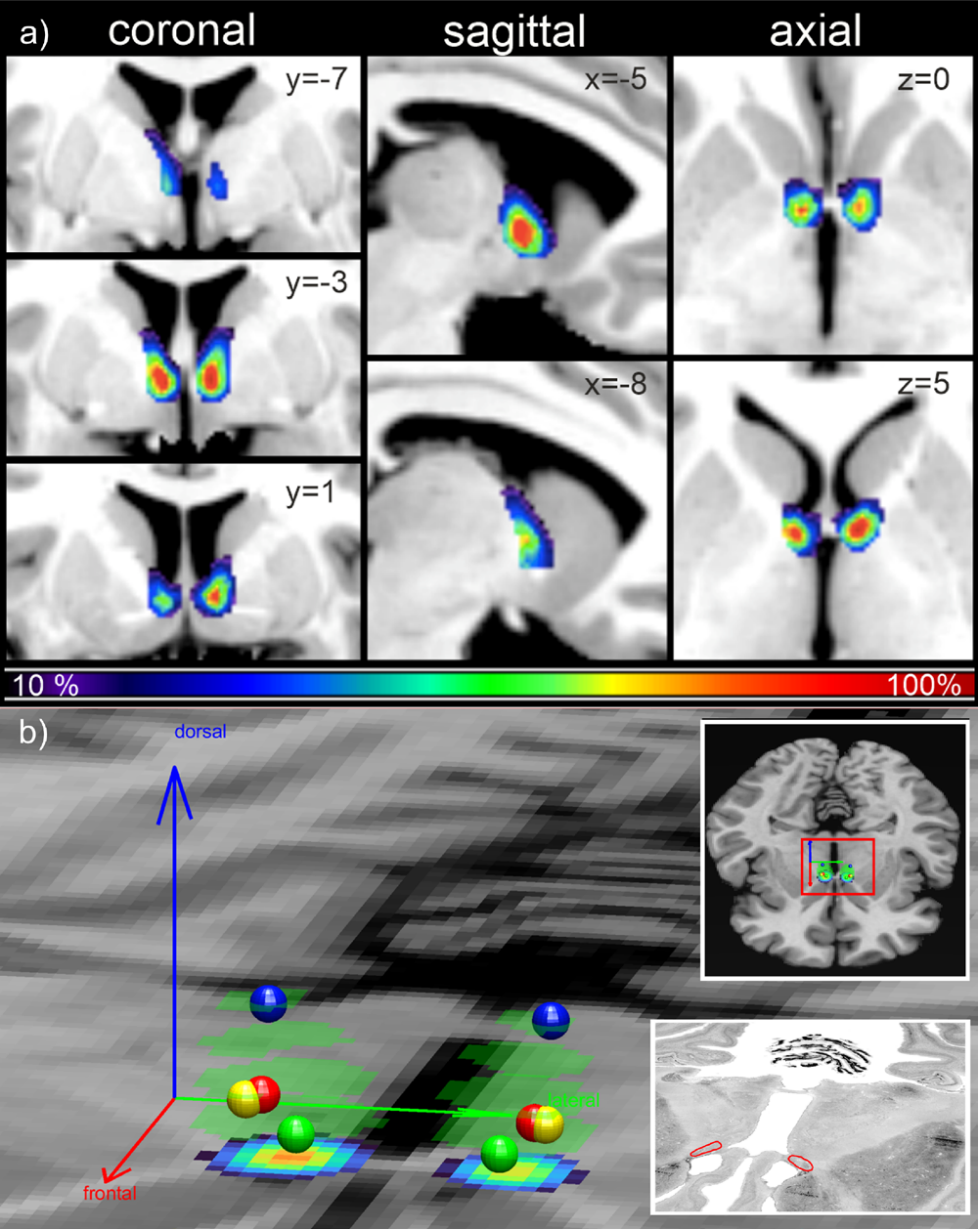
(a) Probabilistic maps of the whole BST of 10 postmortem brains registered to the single-subject MNI Colin-27 template brain in the coronal, sagittal and axial planes. Colors indicate the amount of overlap at certain coordinates in stereotaxic space: Red colors represent high overlap of the 10 brains, blue colors resemble an overlap of only a small number of brains, corresponding to high interindividual probability. (b) Centers of gravity for BST subnuclei from the 20 µm BigBrain annotations visualized on the 1 mm Colin27 template. BSTC is shown in red, BSTD in yellow, BSTM in green, and BSTP in blue. The BST pmap overlays Colin27 at z-level 106, with its upper extent in transparent green. The top right inset shows a full-brain overview, with the main image zooming into the detailed area marked by the red frame. The lower right frame shows the comparable reconstructed horizontal section of the BigBrain model (20 µm in-plane resolution) with red surroundings of the BST as combined structure.

The superimposition of the 10 brains, registered to the MNI Colin-27 single-subject template brain and the *ICBM152casym* (2009) space, resulted in probabilistic maps. These maps provide information about the variability of the BST among the 10 brains given by the probability of the structure for each pixel. The variability of the Bed nucleus is visualized in Figure 7a, with warm colors signifying a high overlap between the 10 brains and cold colors indicating high interindividual variability and therefore less overlap. The elongated and thin shape of the BST and the close location to the lateral ventricle can be recognized at coronal and sagittal planes. The round to oval shape of the cross-section of the BST is depicted in the axial plane.

The probabilistic maps of the BST are publicly available under https://atlases.ebrains.eu/viewer/go/BST_r_MNI152.

### 3.6 The BST and its subdivisions in high-resolution BigBrain space

Since the 1 mm resolution of the Colin-27 template is not sufficient to display the exact position and shape of each of the subdivisions, the BigBrain model was used to calculate centers of gravity at isotropic 20 µm resolution (Amunts et al., 2013). These coordinates were transformed into the Colin-27 reference brain to provide exact anatomical position within the probabilistic map and spatial relationships of each of the subdivisions at the same time (Fig. 7b).

**Fig. 8.**
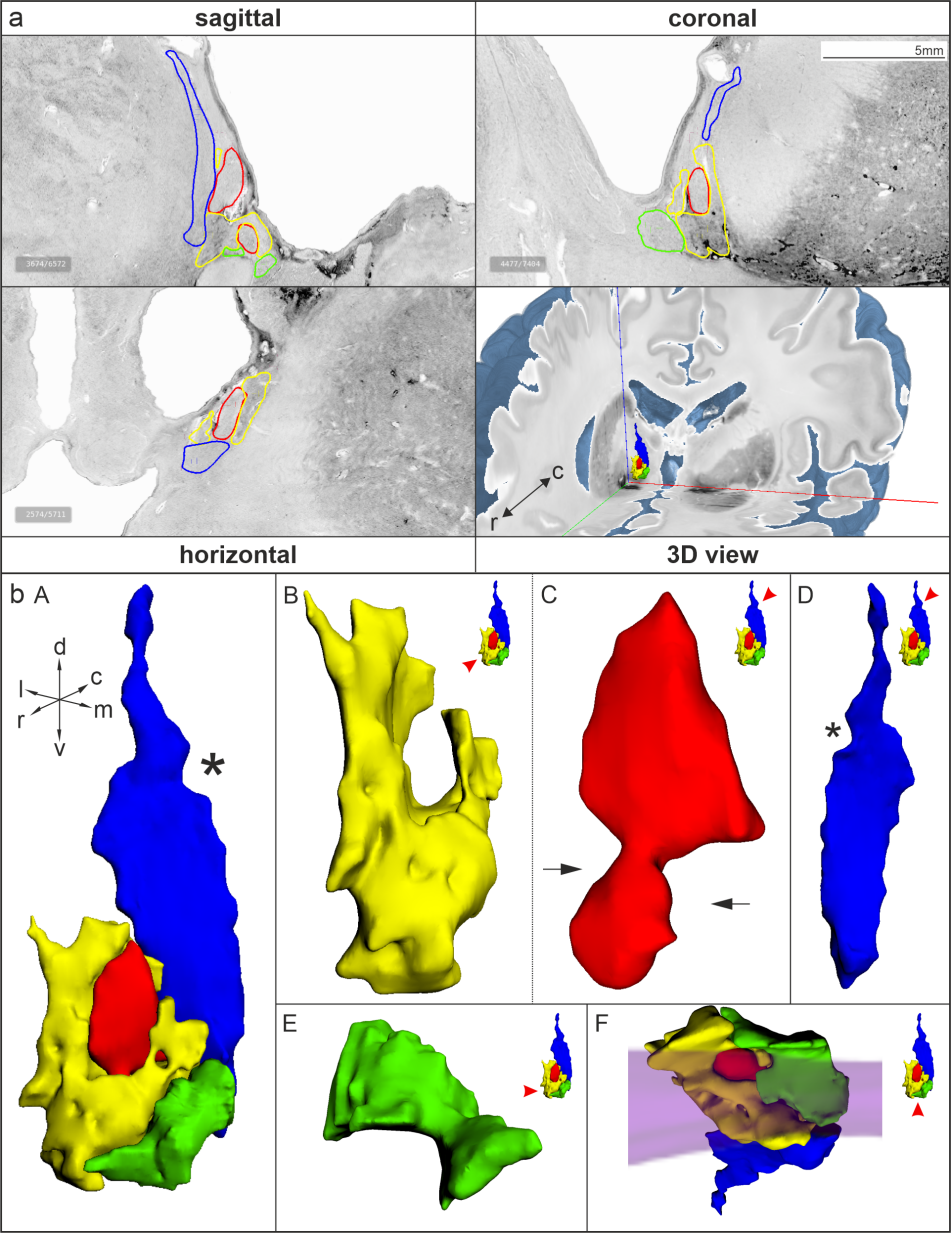
3D-reconstructed Bed nucleus and its subdivisions in the BigBrain (right hemisphere). (a–c) Orthogonal views of the Big Brain (available at EBRAIN: https://atlases.ebrains.eu/viewer/go/bigbrain) and (d) volume rendering using A3D. (e–j) Surface reconstructions of the left Bed nucleus and its four subdivisions. The ventral part of the BSTC (g) is enclosed by the BSTD and narrowed by blood vessels (black arrows in g). The anterior commissure (transparent color in j) is dorsally enclosed by BSTD, BSTM and the ventral tip of BSTC. Asterisks in e and h mark the position of the thalamostriate vein. Color code resembles subdivisions in Figure 3.

The BigBrain model also allows to represent the four subdivisions with high spatial accuracy at 20 µm isotropic and as delineations in 2D images with 1 µm in-plane resolution (https://atlases.ebrains.eu/viewer/go/BST_BigBrain_1um). Figure 8a–d illustrates the shape and location of the Bed nucleus and its subdivisions. In sagittal and coronal plane (Fig. 8a, b) the Bed nucleus seems to be elongated towards its dorsoventral orientation and is clearly dependent of the size and curvature of the lateral ventricle. The horizontal view (Fig. 8c) reveals the oval but slightly narrowed shape at this cross section. Figure 8d shows the location of the Bed nucleus *in situ* within the reconstructed 3D volume from rostral view.

Figure 8e–j displays the high-resolution 3D reconstruction of the Bed nucleus. The whole nucleus in frontal view and the relationship of the subdivisions to each other are visualized in Figure 8e. The second largest subdivision BSTD seems to provide a shell for the BSTC as it is hollowed to a great extent (f). The BSTC itself is oval except for two major indentations on its lower third, deriving from large blood vessels running through the Bed nucleus (g). As this subdivision is almost fully covered by the BSTD, only the dorso-rostral part and the most ventral tip adjacent to the anterior commissure are exposed to the surface of the Bed nucleus. The BSTP is elongated and thin in shape and the largest subdivision of the Bed nucleus (h). Its shape gets even thinner at the dorsal tip when it follows the curvature of the Caudate nucleus and the lateral ventricle. The smallest subdivision, BSTM, is a small, curved structure representing the ventromedial part of the Bed nucleus (i). Looking from lateral view one can see the curvature of the anterior commissure the BSTM and BSTD are enclosing. The subdivisions directly adjacent to the anterior commissure are BSTC, BSTD and BSTM, whereas BSTD and BSTM cover the dorsal part of the anterior commissure (j). Holes and small indented areas indicate blood vessels supplying the Bed nucleus in most cases. The location of the large thalamostriate vein is marked by an asterisk (e and h).

### 3.7 Comparison with previous maps

**Fig. 9.**
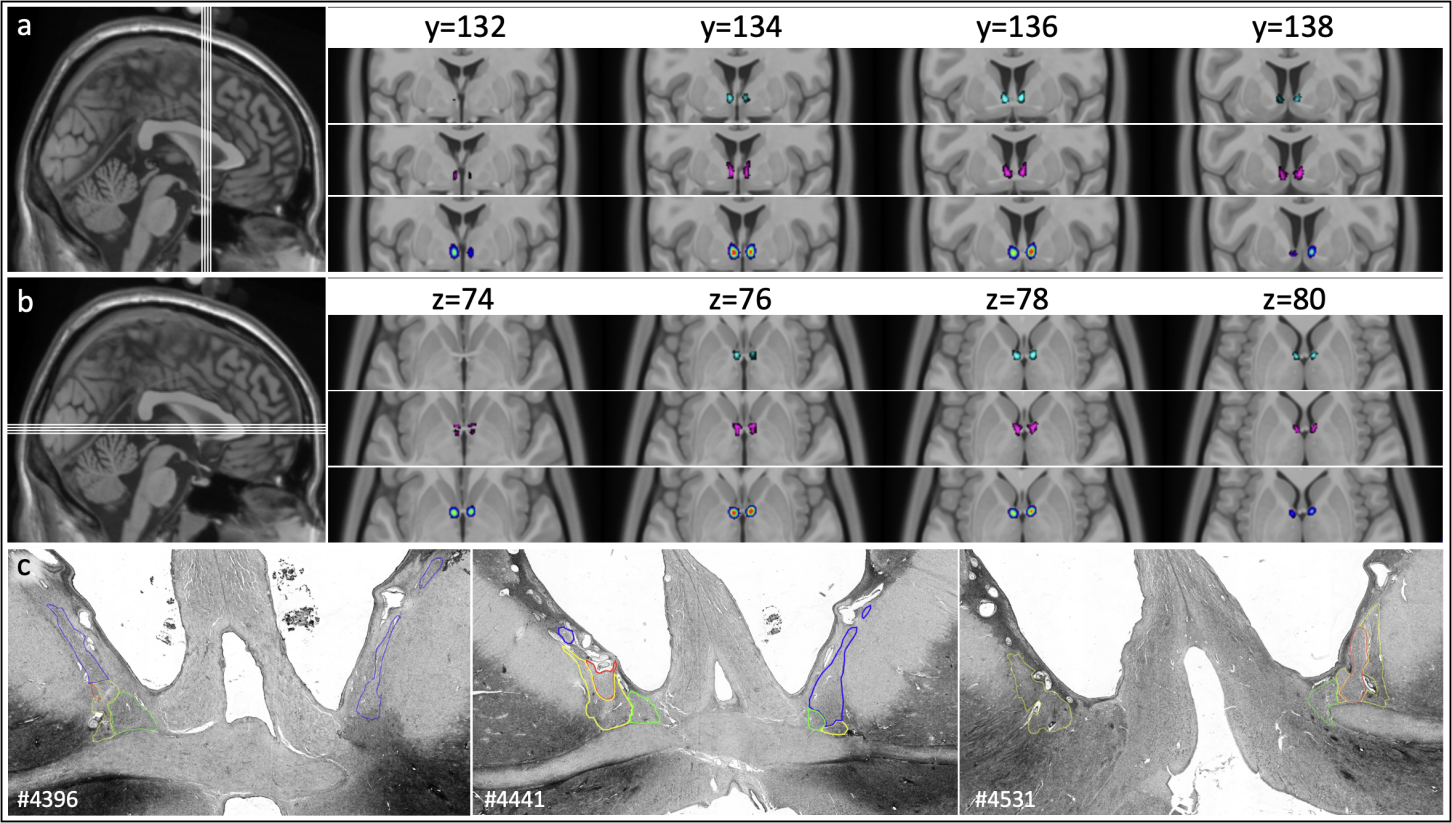
Comparison of probabilistic maps of this study with previous maps. (a) Coronal sections from Theiss et al. (2017) (top row), Sibbach et al. (2024) (middle row) and the current study (bottom row). (b) Analogous images of axial sections. (c) Coronal high-resolution sections and annotations of the present study in the BigBrain model at three different levels, with respective section numbers (from caudal to rostral). Color code: BSTC – red, BSTD – yellow, BSTM – green and BSTP – blue.

The present results of histological mapping were superimposed with MRI-based maps by Theiss et al. (2017) and Sibbach et al. (2024) in the Colin-27 space. Figure 9 shows the maps at four different levels in coronal (a) and axial (b) views. The present maps indicate a more ventral extension of the BST than reported by Theiss et al. (2017) and Sibbach et al. (2024). However, according to our cytoarchitectonic findings there was no distinct ventral subdivision in contrast to the study of Sibbach et al. (2024).

The comparison with the maps in the BigBrain provides an impression of the finer anatomy of the BST and the contributions of the four subdivisions to the map of the entire BST (Fig. 9c).

## 4 Discussion

Four subdivisions of the Bed nucleus of the Stria terminalis were identified and mapped in their full extent, each of them having its own distinct cytoarchitectonic pattern. Maps are part of the Julich-Brain atlas and available via the multilevel Human Brain Atlas under https://ebrains.eu/service/human-brain-atlas to provide microstructural reference data in standard reference space.

### 4.1 The Cytoarchitecture of the BST and its subdivisions

The approach of cytoarchitectonic mapping at microscopical level was used to overcome problems of rather similar cytoarchitectonic pattern of the BST and adjacent Septal nuclei suggesting a considerably larger size of the Bed nucleus in MR images. By gaining results at this high-level precision detailed insights into the shape and exact extent of the Bed nucleus of the Stria terminalis can be obtained.

A ventral subdivision is rather prominent in rat brains (Dong & Swanson, 2006); but appeared as a small structure on single sections in only some brains in the present study. Therefore, it was not possible to reliably map this subdivision over its whole extent, but it was included to the dorsal part of the BST. Especially at levels other than the decussation of the anterior commissure there was no border between a dorsal and ventral subdivision identifiable. These findings might lead to the consideration whether the ventral subdivision could be a remnant in the human BST. A comparison of this ventral subdivision among rodent and primate species should be therefore considered carefully without any additional investigation. For the same reason we cannot confirm the findings of Sibbach et al. (2024), where a prominent ventral subdivision was defined as a ventral subdivision of the BST. We would suggest, also in accordance with literature (Baroncini et al., 2012; Braak & Braak, 1992) that this particular region would rather be part of the hypothalamus. The cytoarchitecture of the latter shows less density of neurons, the neurons are in general larger compared to the ones of the BST. Also, there is a considerable amount of triangular shaped larger neurons in the hypothalamic area, which clearly lack in the BST. It is our understanding that although we appreciate the strength and clarity of these 7T MR-images, it might be a less suitable technique for delineating complex subcortical structures like the basal forebrain and hypothalamus.

The central BST was in this work not considered to be a subdivision of the dorsal part, as suggested by Heimer et al. (1999), but at the same hierarchical level as all other subdivisions, since there was also no hierarchical indication of any other subdivision of the dorsal part, neither was there a structural relationship between the central and anterior subdivision. After careful consideration with previous literature (Baroncini et al., 2012), only the anterior part of the medial BST was mapped. The posterior BSTM could be also considered as perifornical part of the Hypothalamus (Baroncini et al., 2012). The supracapsular part of the BST was considered as part of the posterior part of the BST, as it was not possible to make a reliable cytoarchitectonic distinction between those two subdivisions. In the end, the hierarchical classification of subdivisions was finalized by performing the textural analysis, that was conducted to clarify whether and which subdivisions were discriminant from each other.

The age range of the 10 postmortem brains differed from that of previous mapping studies, for example, Sibbach et al. (2024) or Theiss et al. (2017). It was from 27 to 84 with a mean age of 57.7 years (SD = 19.2) in the current sample. No age-related changes in the BST have been observed. Although an enlargement of the ventricular system has been demonstrated in healthy aging (Currà et al., 2019), which could potentially influence the position of the BST in stereotaxic space, the coordinates of the BST did not show a systematic shift. This negative finding should nevertheless be interpreted in the light of the relatively small sample size of the present postmortem study.

### 4.2 Texture analysis using gray-level co-occurrence matrix

Texture analysis has been applied to further quantify the subdivisions, and to confirm their existence. A major advantage of texture analysis is that any subcortical nucleus can be processed using small image patches to cover polygons regardless of their shape. As other subcortical nuclei, the Bed nucleus lacks of a clear recurrent pattern like the well described lamina in classical cortical areas; therefore well-established tools for the analysis of cortical borders (Schleicher et al., 1999) are not applicable. The texture analysis quantified the architecture of the nucleus in a data-driven manner to enable statistical tests. Another advantage is that data can be directly exported and processed as raw images, that is, digitized histological high-resolution scans as seen under the microscope, resulting in gray-level images at a resolution of 1 µm/px. Therefore, no intermediate steps such as segmentation or thresholding, which might be a possible source of errors, are needed. The herein presented texture analysis applied on histological human brain images is suitable for underlining the distinction of subcortical nuclei and their subdivisions independent of shape or size of the structure and can be applied also on any other brain region (Kedo et al., 2024).

### 4.3 The volume of the BST

The BST has been discussed in terms of a sexual dimorphic structure. While sexual dimorphism of the BST in rodent brains has been confirmed in several studies, data on volumetric differences are much sparser for the human brain, and discussions in the community would be more controversial. The darkly staining region of the posteromedial BST has been described as a sexually dimorphic nucleus with males having a 2.5 times larger nucleus than females, and that this difference may underline sexually dimorphic behavior (Allen & Gorski, 1990). Later, the central subdivision has been reported to be larger in male than female brains, and a role for transsexuality has been assumed based on volume measurements based on the identification of VIP (vasoactive intestinal polypeptide) innervation (Zhou et al., 1995). A more recent study based on data of the Human Connectome Project revealed male-biased sex differences in Guma et al. (2024) and Neudorfer et al. (2020).

The present sample did not show any sex differences, independently of the subdivision. While the present study has higher spatial resolution compared with MR based studies, the BST has a low contrast and is embedded in a densely populated environment, and therefore automatic subdivision segmentation might be challenging. Moreover, the rather small sample size of the present study may result in a bias. Regardless, one might expect that a large sex difference at the level of a factor of 2.5 would be visible in MR images also in a smaller sample, but comparisons with earlier histological studies are not straightforward. The present study corrected for sex differences in overall volume. Nevertheless, if we take the absolute volumes of all BST subdivisions, the subdivisions in male brains are only 1.03 (BSTP) up to 1.18 (BSTM) times larger than those in female brains compared to sample wide differences of the factor of 1.15 for the whole brain volumes.

Comparing the overall size of the Bed nucleus the total size varies in literature: Compares to a volume of 66 mm^3^ in the study of Theiss et al. (2017) study, the Bed nucleus volume was estimated to be 75 mm^3^ in the present study. This slightly larger volume could derive from the fact that we also included the BSTP, a part of the supracapsular Bed nucleus following the curvature of the lateral ventricle. Also, our approach was not limited to a certain distance rostral or caudal from the anterior commissure, but it was with respect to the individual extent of each of the examined brains. Previous studies described the Bed nucleus having the size of a sunflower seed of about 190 mm^3^ (Avery et al., 2014). These annotations, as the ones of Theiss et al. (2017) are based on MR images, where exact borders could not be determined. In standard MR images, it is often assumed that the Bed nucleus reaches the surface of the lateral ventricle, which was contradicted by our findings, as the subventricular zone is always between the ependymal layer of the ventricle and the Bed nucleus (see Fig. 5).

### 4.4 Linking histological maps to in vivo imaging via probabilistic maps

Probabilistic maps in standard template brains have been computed to serve as a new microstructural reference for future structural and functional neuroimaging studies or even neurosurgical applications. By indicating the likelihood that a specific structure is located within a given voxel, these maps provide probabilistic information on the presence of the BST. Because complete, non-thresholded probabilistic maps are always larger than each individual map, this approach explains the overlap between the BST with the anterior commissure in the present study. Previous MR-based masks have defined the borders of the BST using adjacent prominent landmarks, such as the lateral ventricle, anterior commissure, or internal capsule. However, these approaches are less well-suited to account for the more indistinct borders between the BST and neighboring gray matter structures (Avery et al., 2014; Theiss et al., 2017; Torrisi et al., 2019). In particular, gray matter structures located in close proximity—such as the rostral (Nucleus accumbens) and caudal (Hypothalamus) borders of the Bed nucleus—are often considered as fixed distances in millimeters from the decussation of the anterior commissure in these studies (Avery et al., 2014).

The newly developed probabilistic maps can therefore inform functional and structural MR imaging in both healthy subjects and patients, especially in anatomically challenging regions. This is particularly relevant in neurosurgical contexts, where high precision and accurate delineation of anatomical borders are critical for surgical success and treatment outcome. Based on these considerations, we recommend using the mni_icbm152_t1_tal_nlin_asym_09c template as a best practice reference for subsequent analyses.

### 4.5 The BST and its subdivisions in high-resolution BigBrain space

Interactive 3D visualization and segmentation of the BigBrain dataset with Atelier 3D (Borgeat et al., 2007) revealed the localization and extent of the subdivisions, not only in the original coronal cutting plane, as in previous histological studies, but also in the virtual sagittal and horizontal planes. The BigBrain data set is a single model and does not provide information on intersubject variability. However, the comparison of this brain with other postmortem brains of the used sample showed that the BST of the BigBrain is in line with average volume of other brains of four subdivisions (Fig. 6; Table 3). Therefore, it seems to represent the subdivisions in a reasonably adequate way and adds information into the microstructural dimension of the Bed nucleus being inaccessible for other methods. In more detail, our findings of the central subdivision (BSTC) being completely covered by its dorsal counterpart with exception of two sites, that is, dorso-rostrally and ventrally (Fig. 8) opens the discussion for further investigations of connectivity or functional studies. Whether the BSTC’s subdivisions have connections only within the BST itself or interhemispheric connections via the anterior commissure or to other even more distant nuclei remains to be investigated. Answering these questions could also help to understand the specific role of the BSTC and other subdivisions in normal and pathologic anxiety. Of note, the BST in general is also regarded as potential target region for DBS in the treatment of anxiety and obsessive-compulsive disorders or depressive disorders (Feola et al., 2023; Lebow & Chen, 2016; Luyten et al., 2016; Sadeghzadeh et al., 2024; Wang et al., 2025; Winter et al., 2018), even if some results remain to be inconclusive (Fitzgerald et al., 2018, 2024). Additionally, a recent meta-analysis of drug-resistant depression treatment suggested tractography accompanying DBS for patient personalization and more accurate electrode positioning (Sadeghzadeh et al., 2024). Combined with such approaches, our cytoarchitectonic-based maps of the BST’s microstructure will therefore significantly contribute to define the best suited target structure for DBS in those disorders, hereby optimizing potential therapeutic effects and avoiding side effects at the same time. We integrated the fine-grained structure of the BST’s four subdivisions into the BigBrain model, offering a high-resolution map of the BST that provides more detailed information in distinguishing it from neighboring nuclei. Our maps can still be transformed into any other spaces or scales as needed. In accordance with the FAIR guiding principles (Wilkinson et al., 2016), more information can be found in the respective dataset publication in the EBRAINS Knowledge Graph (Brandstetter et al., 2021).

### 4.6 Comparison with previous maps

The maps by Theiss et al. (2017) and Sibbach et al. (2024) were derived from MR images, whereas the present maps are based on serial histological sections stained for cell bodies. Both approaches have inherent advantages and limitations. Histology-based mapping offers high spatial resolution, allowing very narrow sections of the brain that are not visible in MR images because of the much smaller pixel and voxel sizes. This is especially important for the BST, which has a complicated structure with many branches and a large surface.

## 5 Conclusion

This work provides probabilistic and detailed high-resolution maps of the Bed nucleus of the Stria terminalis and its subdivisions in 3D space. A texture analysis confirmed the distinctiveness of the four subdivisions and may serve as input to future modeling and simulation. The current findings contribute to a deeper understanding of the fine-grained parcellation and may support functional and clinical studies dealing with anxiety-related disorders. Furthermore, an accurate mapping of the subdivisions of the Bed nucleus over their full extent is possible only at microstructural level. The maps resulting from this study are publicly available in the 3D Julich-Brain atlas and the EBRAINS Interactive Atlas Viewer.

## Data Availability

The probabilistic maps of the bed nucleus as specific information to data (pre)processing can be found in the EBRAINS knowledge graph under https://doi.org/10.25493/1ZJF-12G (Brandstetter et al., 2021), in accordance with the FAIR guiding principles (Wilkinson et al., 2016). These maps are part of the Julich-Brain Atlas (Katrin Amunts et al., 2020) and openly available via the EBRAINS digital research infrastructure RRID:SCR_019260 (DOI: 10.25504/FAIRsharing.XO6ppp), accessible via https://ebrains.eu/service/human-brain-atlas/. No code was generated in this study.
